# Effect of Calcinated Oyster Shell Powder on Growth, Yield, Spawn Run, and Primordial Formation of King Oyster Mushroom (*Pleurotus Eryngii*)

**DOI:** 10.3390/molecules16032313

**Published:** 2011-03-10

**Authors:** Ung-Kyu Choi, Ok-Hwan Lee, Young-Chan Kim

**Affiliations:** 1 School of Nano-Bioscience and Chemical Engineering, Ulsan National Institute of Science and Technology, Ulsan 689, Korea; E-Mail: cuk8272@unist.ac.kr (U.-K.C.); 2 Department of Food Science and Biotechnology, Kangwon National University, Chuncheon 200, Korea; 3 Institute of Korea Food Research, Seongnam, Kyonggi 463, Korea

**Keywords:** *Pleurotus eryngii*, fruiting body, calcinated oyster shell powder, sawdust culture media

## Abstract

This study was conducted to evaluate the calcium (Ca) absorption efficacy of king oyster mushroom (*Pleurotus eryngii*) grown on sawdust medium supplemented with Ca-sources, including oyster shell powder, and to determine the efficacy of oyster shell powder as a calcium supplement on growth, yield, spawn run and primordial formation of *P. eryngii*. Optimum calcination of oyster shell powder was achieved at the temperature of 620.56 °C. A 1% supplementation of oyster shell powder in sawdust medium did not suppress the mycelial growth of *P. eryngii*. Also the supplementation of 2% calcinated oyster shell powder to sawdust medium potentially increased the calcium content up to a level of 315.7 ± 15.7 mg/100 g in the fruiting body of *P. eryngii*, without extension of duration of spawn run and the retardation of the days to primordial formation. These results suggest that the shellfish by-products, including oyster shell powder, can be utilized to develop calcium enriched king oyster mushrooms.

## 1. Introduction

King oyster mushroom (*Pleurotus eryngii*) grows on a wide variety of solid lignocellulosic substrates [1,2,3], and it has become one of the most popularly cultivated mushrooms in the World [4]. *P. eryngii* mushroom is taxologically related to *Basidomycotina*, *Agricales*, *Pleurotaceae* and *Pleeurotus* [5], which are characterized by bell-shaped fruit bodies bearing a gill hymenophore as well as for being commonly cultivated in Europe, Middle East, North America, and in parts of Asia. *P*. *eryngii*, highly valued for its superior texture, flavor and nutritional quality, is commonly known as king oyster mushroom or king trumpet mushroom. Recently *P. eryngii* has been cultivated in plastic bottles manufactured for mushroom cultivation. The value of *P*. *eryngii* as an exportable item is very high due to its lesser moisture content and it is expected to become an important potential source of agricultural revenue [6,7].

Mushroom cultivation is a useful approach to treat solid agricultural and industrial wastes in both developed and developing countries [8,9]. In cultivation, insoluble calcium salts are added to increase pH to neutrality and thereby probably reducing the bacterial contamination, and to increase aeration by aggregation, improving the texture and porosity of the compost [10,11]. Recent evidence shows that irrigation with water containing calcium chloride improves the postharvest storage of mushroom by reducing the surface bacterial population [12].

Several studies have been conducted to investigate the heavy metals absorption efficacy of *Pleurotus eryngii*, *Hypsizygus marmoreus*, *Pholiota nameko*, *Flammulina velutipes* and *Ganoderma lucidum* from various Ca-sources [13,14,15,16]. In general, the total amount of calcium content in the mushrooms remains lesser than that of vegetables [17,18]. As a part of efforts to enrich the mineral content in the mushroom, Tabata and Ogura [19] reported that potato sucrose agar (PSA) and sawdust media with supplementation of 1% Ca salts increased the Ca content of fruiting bodies in *H. marmoreus*. The calcium absorption efficacy of *P. ostreatus* and *P. nameko* in PSA and sawdust media has also been well documented [20].

The calcium absorption efficacy of king oyster mushroom (*P. eryngii*) in sawdust medium supplemented with calcium salts as well as the effect of calcium content on mycelial growth of *P. eryngii* on potato dextrose agar (PDA) medium have been reported previously [21]. Furthermore, we previously reported [22] that treatment with a Ca-source such as calcinated starfish powder potentially increased the Ca content in the fruiting body of *P. eryngii*. However, the effect of shellfish by-products, including calcinated oyster shell powder, on growth, yield, spawn run, and Ca content of *P. eryngii* has not been investigated. Because oyster shell is not only good calcium enriched natural products but also a freely available waste, in the present study, we investigated the effect of various levels of calcinated oyster shell powder on the mycelial growth of *P. eryngii* supplemented in sawdust media and determined its effect on yield, spawn run and primordial formation of *P. eryngii*.

## 2. Results and Discussion 

### 2.1. Calcination Characteristics of Oyster Shell Powder

**Figure 1:**
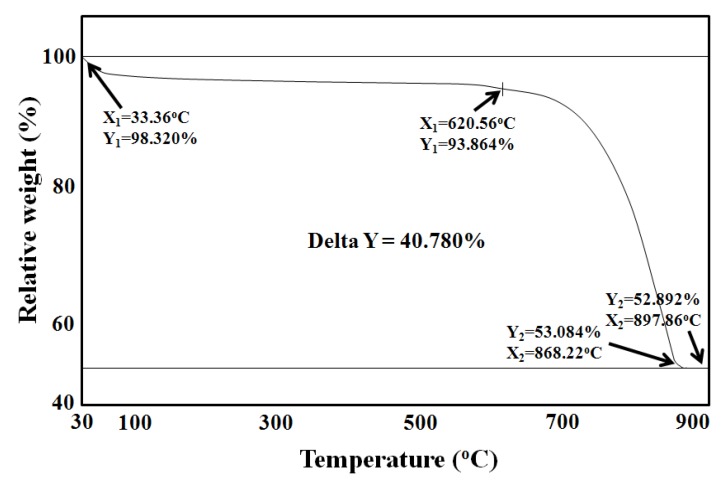
Thermal gravimetric analysis (TGA) profile of raw oyster shell powder due to thermal decomposition.

Thermal gravity analysis (TGA) was used to determine the calcination characteristics of the oyster shell powder. The TGA profile of raw oyster shell powder is shown in Figure 1. There was change in the weight of the oyster shell as the temperature was raised from 30 to 900 °C due to calcinations, which started at 620.56 °C, however, the complete calcination of oyster shell powder resulting in a phase-change of the sample was only achieved at 897.86 °C. A major component of the oyster shell powder was identified as pure CaCO_3_ at the temperature of 620.56 °C. The humidity and organic content removal was achieved below 620.56 °C. When temperature was raised from 620.56 to 897.86 °C, CaCO_3_ and CaO were identified as the major components of the oyster shell powder. A major component of the oyster shell powder at temperatures above 868.22 °C was identified as pure CaO. It was assumed that most of the oyster shell was converted to CaO by final pyrolysis at temperatures higher than 868.22 °C. Thus, this implies that the optimum temperature of calcination of oyster shell was achieved at the temperature range around 620.56 °C.

### 2.2. Effect of Oyster Shell Powder on the Mycelial Growth of P. Eryngii

**Figure 2:**
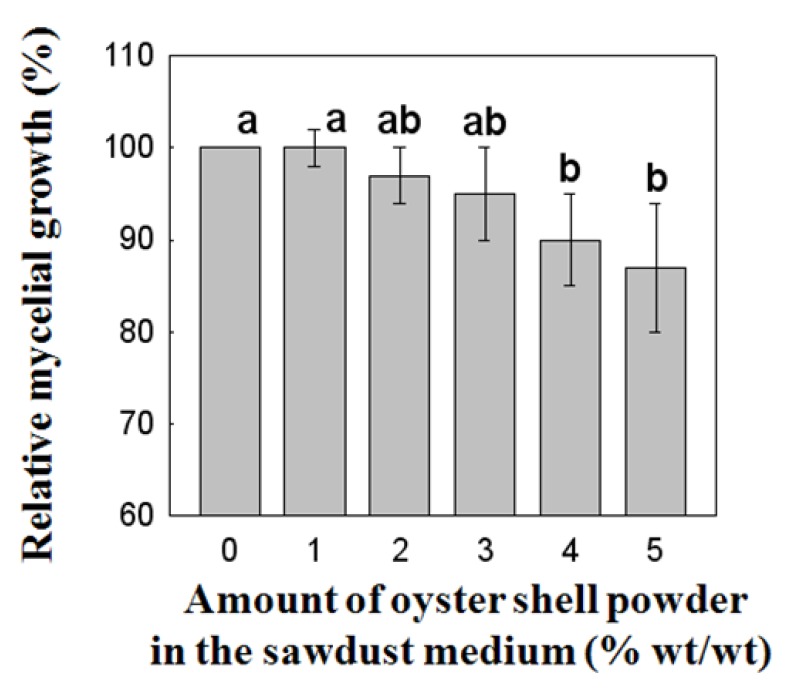
Relationship between the content of calcinated oyster shell powder in sawdust substrate and mycelia growth of *P. eryngii* (data point are means of 5 replicates). *P. eryngii* was cultivated at 25 °C, pH 7.0 for 48 h. Values with different letters are significantly different among experimental groups at p < 0.05 by Duncan’s multiple range test.

Figure 2 shows the influence of oyster shell powder addition in sawdust medium on the growth of *P. eryngii*. Mycelial growth of *P. eryngii* decreased as the amount of oyster shell powder in the sawdust substrate was increased from 1 to 5% (wt/wt), but a significant decrease was observed with increments above 4% oyster shell powder addition. Therefore, we may conclude that while addition of more than 4% oyster shell powder in sawdust medium significantly suppressed the growth of mycelia (Figure 2), the mycelial growth was not changed significantly by the supplementation of 1 to 3% of oyster shell powder contents in the sawdust medium. It can be estimated that the small concentration dependency of oyster shell powder on mycelial growth might be attributable to its low solubility. These results is supported by a study [19] showing that the mycelial growth of *H. marmoreus* cultured on potato sucrose agar (PSA) medium was slightly increased by augmentation with 0.5% calcium carbonate as compared to the control and complete suppression was achieved by 5% addition in this medium. When cultivating *P. eryngii* in both potato dextrose agar (PDA) and sawdust media supplemented with Ca salts, it was observed that addition of Ca phosphate and Ca carbonate to sawdust media did not affect the growth, whereas Ca sulfate addition suppressed the mycelial growth and this might be affected by the varied pH levels of media [21].

### 2.3. Effect of Oyster Shell Powder on Duration of Spawn Run and Time to Primordial Formation of P. eryngii

**Figure 3:**
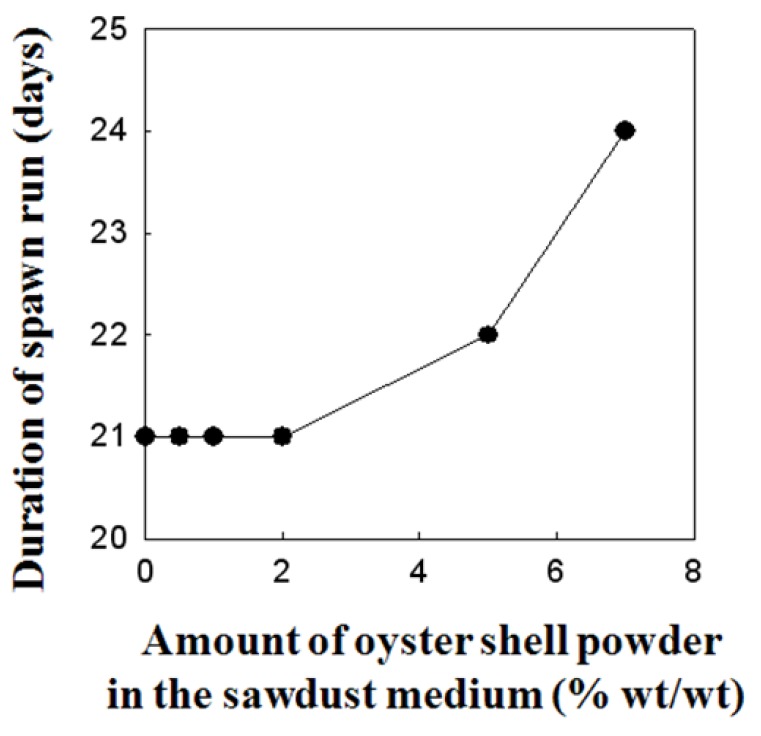
Effect of supplanting sawdust substrate with oyster shell powder in sawdust medium as a Ca-source on duration of spawn run of *Pleurotus eryngii*.

**Figure 4:**
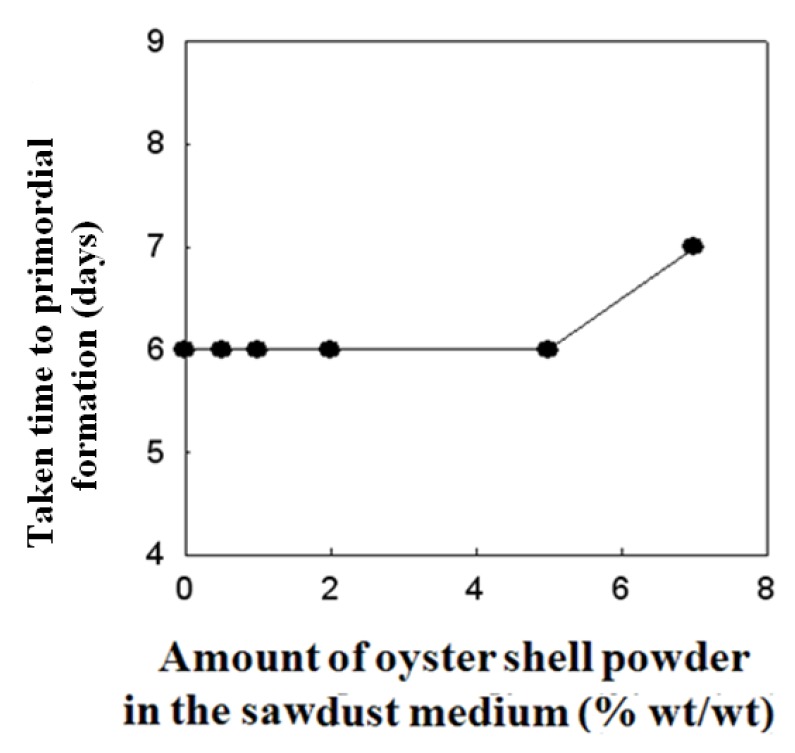
Effect of supplanting saw dust substrate with oyster shell powder in sawdust medium as a Ca-source on the taken time to primordial formation of *Pleurotus eryngii*.

The effects of oyster shell powder supplementation on duration of spawn run and time to primordial formation of *P. eryngii* are shown in Figure 3 and Figure 4. Increasing the oyster shell powder content of the sawdust substrate by up to 2% did not significantly affect either the duration of spawn run and the time taken to primordial formation, but the addition of more than 2% of oyster shell powder to sawdust medium suppressed the growth of *P. eryngii*. Mushrooms, including *P. eryngii*, are known to be excellent agricultural products having various functional materials in spite of their disadvantage of having lower calcium content than vegetables. Several studies revealed the absorption efficiency of metal ions from the culture media that occurs during the inorganic absorption of mushrooms including *P. ostreatus*, *H. marmoreus*, *P. nameko*, *F. velutipes* and *G. lucidum* [21]. In our previous study [22], conducted on *Pleurotus eryngii* using commercially available calcium salts, we found a strong correlation with our recent findings [22], confirming the calcium absorption efficacy of *P. eryngii* mushroom in sawdust medium supplemented with calcinated starfish powder as well as the effect of calcinated starfish powder on growth, yield, spawn run and primordial formation of *Pleurotus eryngii* [22]. It is also expected that numerous factors in the environment (e.g., pH, moisture content, climate, *etc.*) can additionally affect the absorption efficiency of calcium content into fruiting bodies of *P. eryngii*. Based on the above findings, it is expected that passive transportation pathways of calcium uptake either via a non-specific channel in plasmalemma and further diffusive transport along the hyphae or extra-cellular transport from the medium through the inter-hyphal cavity into the fruiting body may exist. These observations highlight the distinctiveness of the mechanisms involved in the uptake and absorption of calcium content in higher classes of mushrooms. However, the relevance of calcinated oyster shell powder involvement in any of these pathways clearly requires further investigation.

### 2.4. Effect of Oyster Shell Powder on Calcium Content of the Fruiting Body

**Figure 5:**
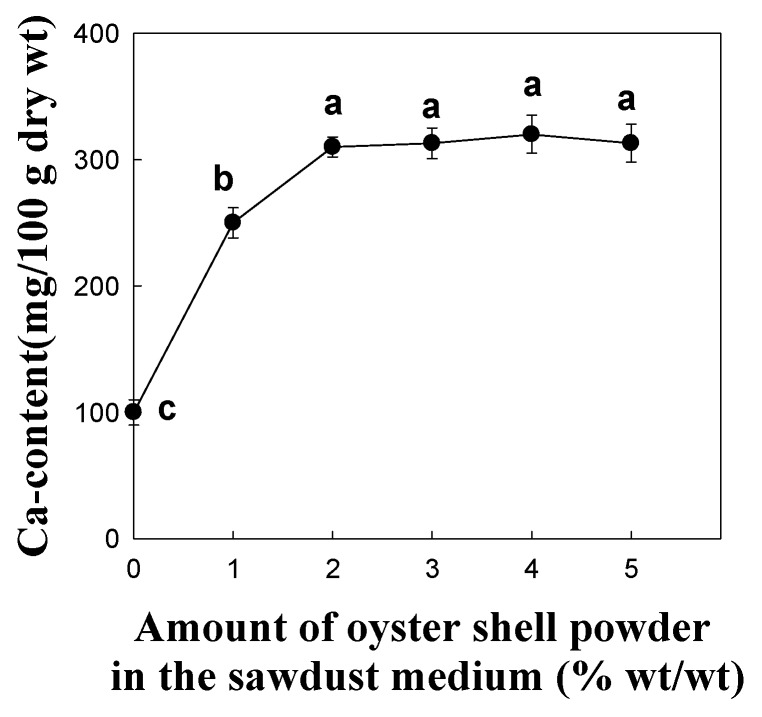
Effect of calcinated oyster shell powder on calcium content of *P. eryngii* fruiting body. Values with different letters are significantly different among experimental groups at p < 0.05 by Duncan’s multiple range test. Data points are means of five replicates.

The calcium absorption efficacy of *P. eryngii* fruiting bodies is shown in Figure 5. As the amount of oyster shell powder in the substrate was increased from 0 to 5%, the calcium content of the fruiting body increased rapidly to a plateau of more than 2% of oyster shell powder in the substrate. The plateau concentration of calcium in the fruiting bodies was more than 300 mg/100 g. *P. eryngii* cultivated on sawdust medium without calcium source showed 101.3 ± 3.9 mg/100 g of calcium content in fruiting body (Figure 5). Increasing the oyster shell supplementation in the sawdust substrate to 1 and 2% increased the calcium content in the fruiting body to a level of 253.7 ± 14.5 mg/100 g and 315.7 ± 15.7 mg/100 g as compared to control, respectively. Addition of calcium source for up to 10 days showed minor differences of calcium content in the fruiting bodies of *P. eryngii*, whereas after 15 days, differences in calcium content became significant (data not shown). The calcium content in the fruiting bodies of *P. eryngii* tended to increase with increasing content of calcium source added to the culture media, although addition of more than 3% of oyster shell powder significantly reduced the fruiting body yield, suggesting a diminished industrial usefulness (Figure 3). We recently reported that supplementation of calcinated starfish (1%), another Ca-source, markedly enhanced the calcium contents in the fruiting bodies to a level of 256.0 ± 16.3 mg/100 g as compared to control (70.0 ± 16.3 mg/100 g) [22]. 

**Figure 6:**
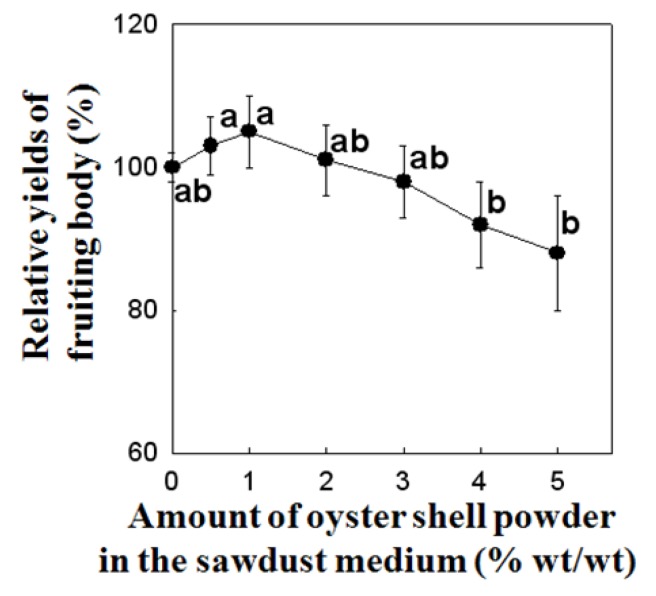
Effect of oyster shell powder in sawdust medium as a Ca-source on relative fruiting body yield of *Pleurotus eryngii*.Values with different letters are significantly different among experimental groups at p < 0.05 by Duncan’s multiple range test. Data points are means of five replicates.

It was found that the calcium contents of *P. eryngii* were increased more than three-fold by addition of 2% oyster shell powder as a calcium source. Further the yield of fruiting bodies did not differ significantly after addition of 2% calcinated oyster shell powder to the sawdust medium compared to control (Figure 6). 

Thus, it can be expected that 2% addition of oyster shell powder to the sawdust medium is the desirable level to produce good quality of *P. eryngii* with a higher calcium content. According to cultivation time, the variations of calcium contents in the fruiting body of *P. eryngii* were found to be similar to those of the calcinated oyster shell powder addition.

## 3. Experimental

### 3.1. Strain

The strain of *P. eryngii*, originally cultured at a mushroom farm (Mushheart Co., Anseong, Korea), was purchased from the National Institute of Agricultural Science and Technology, Republic of Korea.

### 3.2. Preparation of Calcinated Powder of Oyster Shell

The oyster shells (500 kg) were collected from East Coast of Republic of Korea. The oyster shells were cut into the pieces (10 mm × 10 mm), soaked in distilled water for 24 h to remove the salt layer and left to dry naturally. To remove impurities and interfering materials such as organics and salts, the sample was rinsed several times with deionized water. The sample was dried at 100 °C for 24 h in the dry oven. Dried oyster shell powder was pulverized and subjected to calcination. Finally, samples having 40–100 mesh separated with a vibration selector were used for analysis of the calcium absorption efficacy of *Pleurotus eryngii*.

### 3.3. Measurement of Calcination Characteristics of Oyster Shell Powder

In order to measure the calicination characteristics of oyster shell powder, a thermal gravimetric analyzer (TGA-951 DuPont Instrument Co., USA) was used. A portion of the dried oyster shell powder previously purified from interfering organic salt material was loaded into the TGA analyzer and the pyrolysis up to 900 °C at a rate of 40 °C/min was performed with continuous injection of N_2_.

### 3.4. Effect of Oyster Shell Powder on Mycelial Growth of P. Eryngii

A mixture of sawdust and rice bran (4:1 w/w) was supplemented with 0, 1, 2, 3, 4 and 5% oyster shell powder, followed by addition of tap water (10.9 ppm Ca content) to give a moisture content of 65%. The mixture was homogenized. Thirty grams of the medium tightly packed into a Petri dish was autoclaved at 121 °C for 50 min and allowed to cool for 6 h. An agar mycelial disc of previously grown *P. eryngii* (from 7 day old culture) was picked using a sterile cork borer and placed upside down in the center of each plate supplemented with oyster shell powder. The plates were then incubated for 5 days at 25 °C, the diameter of the mycelial growth was determined [21]. All experiments were conducted in 5 replicates.

### 3.5. Analysis of Ca Contents in Fruiting Bodies of P. Eryngii

The calcium contents in fruiting bodies of *Pleurotus eryngii* were analyzed by the atomic absorption spectroscopy analytical method as described previously [23].

### 3.6. Effect of Oyster Shell Powder on Fruiting Body Harvest in Sawdust Medium

Sawdust medium supplemented with 0, 1, 2, 3, 4 and 5% of oyster shell powder was prepared and filled in a heat-resistant polypropylene bottle (approximate capacity, 850 mL). A cavity (1.5 cm diameter × 10 cm deep) was made in the center of the medium for the inoculation. The sterilized medium was inoculated with 10 mL of liquid spawn and incubated at 25 ± 1 °C in the dark. When the spawn had grown throughout the medium, the culture was subjected to kinkaki treatment for the removal of mycelial surface to induce budding [24]. The culture was then transferred to a culture room maintained at 15 ± 2 °C with 80 ± 5% of relative humidity. The fruiting bodies of *Pleurotus eryngii* were harvested and dried in an oven at 60–70 °C for 72 h. A homogeneous sample was prepared by grinding the dried fruiting bodies in a stainless-steel mill.

## 4. Conclusions

This study revealed the calcium absorption efficacy of *P. eryngii* by the supplementation of raw oyster shell powder as calcium source, resulting in higher calcium contents in the fruiting bodies of *P. eryngii* up to 1% addition of the oyster shell powder without any changes of yield, duration of spawn run and taken time to primordial formation. Thus, these findings reinforce the suggestion that the nutritional value of *P. eryngii* mushroom as a functional food material can be greatly improved by increasing the normal sub-optimal calcium content levels. Further research will be conducted on *P. eryngii* using various natural calcium enriched sources such as crab-shell, shrimp-shell, clam-shell and starfish shell. In fact, if these resources are going to be developed as practical medium additives for *P. eryngii*, the functional value of *P. eryngii* mushroom consumption is expected to increase considerably, as the calcium content deficiency of king oyster mushroom was remedied with addition of oyster shell powder without any changes of yield, duration of spawn run and taken time to primordial initiation.
